# Supplementary material for: DFT, ADMET and Molecular Docking Investigations for the Antimicrobial Activity of 6,6′-Diamino-1,1′,3,3′-tetramethyl-5,5′-(4-chlorobenzylidene)bis[pyrimidine-2,4(1H,3H)-dione]
Source: Molecules. 2022 Jan 18;27(3):620. doi: 10.3390/molecules27030620 (PMC8839838; doi:10.3390/molecules27030620)
Supplement: Supplementary file 1 [file molecules-27-00620-s001.zip › molecules-1548728-supplementary.pdf]

The vibrational frequency results obtained from the optimized structural parameters by using the same level of calculations (B3LYP method with 6-311G** basis set).

The entire vibrational assignments of wave numbers were made on the basis of potential energy distribution (PED) by VEDA 4 program

Mode No.	Type	Bond	Calculated Frequency	PED%	Mixed Mode	PED%
1	STRE	NH	f3677	19	f3557	80
2	STRE	NH	f3677	80	f3557	19
3	STRE	NH	f3678	93		
4	STRE	NH	f3574	94		
5	STRE	CH	f3192	99		
6	STRE	CH	f3206	98		
7	STRE	CH	f3200	95		
8	STRE	CH	f3156	96		
9	STRE	CH	f3086	-96		
10	STRE	CH	f3024	90		
11	STRE	CH	f3171	93		
12	STRE	CH	f3182	87	f3059	13
13	STRE	CH	f3117	100		
14	STRE	CH	f3182	-13	f3059	86
15	STRE	CH	f3180	86	f3058	13
16	STRE	CH	f3168	92		
17	STRE	CH	f2970	100		
18	STRE	CH	f3116	-99		
19	STRE	CH	f3180	-13	f3058	86
20	STRE	CH	f3083	-94		
21	STRE	CH	f3020	88		
22	STRE	OC	f1719	-72		
23	STRE	OC	f1766	-76		
24	STRE	OC	f1728	-70		
25	STRE	OC	f1771	72		
26	STRE	CC	f1678	-14	f1646	43
27	STRE	CC	f1619	49		
28	STRE	CC	f1443	10	f1321	-16
29	STRE	CC	f1443	18	f1391	18
30	STRE	CC	f1632	-48	f1202	-19
31	STRE	CC	f1100	66		
32	STRE	CC	f1632	15	f1615	42

33	STRE	NC	f1051	-11		
34	STRE	NC	f1489	13	f1219	17
35	STRE	NC	f1462	14	f1388	20
36	STRE	NC	f1489	-13		
37	STRE	NC	f1209	22		
38	STRE	NC	f1487	13	f1285	-11
39	STRE	NC	f1057	-38		
40	STRE	NC	f1072	-12	f1061	36
41	STRE	NC	f468	-11		
42	STRE	NC	f1051	10	f470	-15
43	STRE	NC	f1274	11	f914	13
44	STRE	NC	f942	14		
45	STRE	NC	f1385	14	f517	10
46	STRE	CC	f1285	15	f1072	11
47	STRE	NC	f524	-11		
48	STRE	CC	f1274	10		
49	STRE	CC	f1198	16	f183	11
50	STRE	ClC	f671	11	f455	32
51	BEND	CCC	f1615	17		
52	BEND	CNC	f434	60		
53	BEND	CNC	f1385	13		
54	BEND	HNC	f1164	57		
55	BEND	HNH	f1683	67	f1619	11
56	BEND	HNC	f1168	-45		
57	BEND	HNH	f1678	53	f1646	26
58	BEND	HCC	f1443	-23	f1391	-11
59	BEND	HCC	f1519	54	f1198	15
60	BEND	HCC	f1632	17	f1202	-51
61	BEND	HCC	f1321	72		
62	BEND	HCH	f1522	-12	f1460	11
63	BEND	HCH	f1522	22	f1489	-27
64	BEND	HCH	f1515	-60	f1149	25
65	BEND	HCH	f1501	-36	f1460	-17
66	BEND	HCH	f1501	37	f1460	-17
67	BEND	HCH	f1507	57	f1260	10
68	BEND	HCH	f1462	41	f1460	10
69	BEND	HCH	f1514	-57	f1149	-23
70	BEND	HCC	f1362	64		
71	BEND	HCH	f1499	61	f1157	-27
72	BEND	HCH	f1508	53	f1499	-11
73	BEND	HCH	f1425	10	f1424	57

74	BEND	HCH	f1524	-28	f1487	-25
75	BEND	CCN	f1489	12		
76	BEND	NCO	f422	-48		
77	BEND	CCN	f682	23		
78	BEND	CCC	f758	-13		
79	BEND	CCC	f183	20		
80	BEND	CCC	f182	-16	f169	22
81	BEND	NCO	f747	-38		
82	BEND	CCC	f1519	12	f1030	74
83	BEND	CCC	f1615	11	f650	-40
84	BEND	NCN	f517	15		
85	BEND	CNC	f347	12	f343	22
86	BEND	NCN	f517	10		
87	BEND	CCO	f438	52	f376	11
88	BEND	CCN	f1487	10	f468	14
89	BEND	CCN	f416	21	f414	-11
90	BEND	CNC	f470	15		
91	BEND	CNC	f468	26		
92	BEND	CCO	f706	10	f626	10
93	BEND	CNC	f414	-25		
94	BEND	CNC	f419	-19	f418	-37
95	BEND	CNC	f347	18	f343	-12
96	BEND	CCC	f143	-16	f27	13
97	BEND	CCC	f33	17		
98	BEND	CCC	f201	21		
99	BEND	ClCC	f343	-17	f333	23
100	TORS	HNCN	f414	16	f318	16
101	TORS	HNCN	f566	-68		
102	TORS	HNCN	f595	60		
103	TORS	HNCN	f422	-10	f376	39
104	TORS	HCCC	f975	62	f732	-10
105	TORS	HCCC	f968	32	f956	-24
106	TORS	HCCC	f832	94		
107	TORS	HCCC	f855	21	f830	48
108	TORS	HCNC	f1260	18	f1057	-10
109	TORS	HCNC	f1515	-18	f1149	69
110	TORS	HCNC	f216	56	f169	-15
111	TORS	HCNC	f1501	18	f1157	-34
112	TORS	HCNC	f1157	35	f109	-20
113	TORS	HCNC	f1507	-17	f1298	10
114	TORS	HCNC	f1157	-12	f107	-11

115	TORS	HCNC	f1514	13	f1149	33
116	TORS	HCCC	f1443	18	f1391	-50
117	TORS	HCNC	f1499	-20	f1157	56
118	TORS	HCNC	f1508	-14	f1302	13
119	TORS	HCNC	f1149	-41	f213	22
120	TORS	HCNC	f1524	-10	f1267	-14
121	TORS	CCCC	f14	56		
122	TORS	CCNC	f107	45		
123	OUT	CCCC	f524	21	f293	-10
124	TORS	CCCC	f975	-12	f419	56
125	TORS	CCCC	f143	-11	f48	19
126	TORS	CNCN	f33	-49		
127	TORS	CCCC	f40	66		
128	TORS	CCNC	f132	-11	f128	-18
129	TORS	CNCN	f90	39		
130	TORS	CCNC	f109	28	f93	23
131	TORS	CCCC	f36	62		
132	TORS	CNCN	f178	-11	f132	-28
133	TORS	CNCN	f75	53		
134	TORS	CCCC	f48	39	f27	31
135	OUT	OCNC	f787	50		
136	OUT	OCNC	f778	-52	f706	14
137	OUT	ONNC	f763	70		
138	OUT	ONNC	f765	66	f758	13
139	OUT	ClCCC	f347	11	f326	-11
140	OUT	NCNC	f700	32	f690	-21
141	OUT	NNCC	f706	-29	f700	13
142	OUT	CCCN	f178	29	f132	-15
143	OUT	CCCN	f270	-14	f267	-19
144	OUT	CCCN	f270	-25	f267	33
145	OUT	CCCN	f216	-15	f128	17
146	OUT	CCCC	f293	11		
147	TORS	CCCC	f732	48	f27	11

Crystallographic data

Chemical formula	$C_{19}H_{21}ClN_6O_4$
Molecular Weight (g/mol)	432.87
space group	Orthorhombic, $P2_12_12_1$
Lattice Parameters (Å)	
a	11.5365
b	14.5935
c	23.2102
Volume (Å^2)	3907.62
Z	8
μ (mm^{-1})	0.24
Temperature (K)	293
Radiation type	Mo Kα
Crystal size (mm)	0.40 x 0.28 x 0.16
